# Vision loss, disability and multimorbidity among older people in Andhra Pradesh and Telangana in South India: the longitudinal eye health, ageing and disability study (LEADS) – study protocol

**DOI:** 10.1136/bmjph-2025-004598

**Published:** 2026-06-09

**Authors:** Srinivas Marmamula, Vidya Paramparambath, Thirupathi Reddy Kumbham, Vinitha Mingi, Anand Sagar Avula, Uday Kishore Pusuluru, Venkateswara Rao Pilla, Soumya Sri Ambaragonda, Shravya Maddela, Shiva Kumar Chakali, Kavitha Kummari, Anjali Boravelly, Sai Krishna Sirimalla, Swetha Krishnan, Charulatha Sekar, Abhinaya Mohan, Lakshmi Narasamma Kambhapu, Joshua R Ehrlich, David E Bloom

**Affiliations:** 1 Allen Foster Community Eye Health Research Centre, Gullapalli Pratibha Rao International Centre for Advancement of Rural Eye care, L V Prasad Eye Institute, Hyderabad, Telangana, India; 2 Wellcome Trust/Department of Biotechnology India Alliance, L V Prasad Eye Institute Allen Foster Community Eye Health Research Centre, Hyderabad, Telangana, India; 3 Brien Holden Institute of Optometry and Vision Science, Hyderabad Eye Research Foundation, Hyderabad, Telangana, India; 4 Institute for Social Research, University of Michigan, Ann Arbor, Michigan, USA; 5 Department of Ophthalmology and Visual Sciences, University of Michigan, Ann Arbor, Michigan, USA; 6 Harvard T H Chan School of Public Health, Boston, Massachusetts, USA

**Keywords:** Epidemiologic Research Design, Public Health, Community Health

## Abstract

**Introduction:**

Vision impairment (VI), multimorbidity and disabilities are common among older populations. Although the prevalence of VI and its associations are frequently reported, the incidence and risk factors are not well documented in India. The Longitudinal Eye Health, Ageing and Disability Study (LEADS) aims to: (a) determine the prevalence, causes, risk factors and impact of VI; (b) explore the associations between VI and sociodemographic risk factors, systemic health conditions (multimorbidity), physical performance, hearing, depression, social connectedness and cognitive function; and (c) evaluate the impact of eye interventions such as cataract surgery and refractive correction (for distance and near) on these conditions. Additionally, (d) to assess the annual incidence of and risk factors for VI among older people in the community.

**Methods and analysis:**

Individuals aged ≥60 years are selected from seven districts across three regions in the states of Andhra Pradesh and Telangana in Southern India. A two-stage cluster random sampling method is used to enrol participants. Trained field investigators administered a set of questionnaires, including personal and sociodemographic information, ocular and systemic history, cognition, depression, hearing, falls and fear of falling. The Short Physical Performance Battery is used to assess physical performance. A comprehensive eye examination is conducted in makeshift clinics set up nearby. The examination included unaided and aided visual acuity for distance and near, using minimum logarithm of resolution charts, manual and auto refraction, slit lamp biomicroscopy and anterior and posterior segment imaging using a non-mydriatic fundus camera. The individuals will be examined at baseline (wave I) and then at a follow-up visit planned after 18–24 months (wave II).

**What is already known on this topic**

- Vision impairment is highly prevalent in the older population in India. Vision impairment impacts several dimensions of health and well-being in the older population.

**What this study adds**

- First comprehensive eye health study investigating the incidence, the impact of vision impairment on health and well-being and impact of eye health interventions such as cataract surgery and spectacles for refractive errors in the older populations in the states of Andhra Pradesh and Telangana in south India.
- The protocol described in this paper can be used to replicate and conduct similar studies in low- and middle-income countries.

**How this study might affect research, practice or policy**

- As this study represents over 92 million population in the states of Andhra Pradesh and Telangana, it is envisaged to provide valuable data on eye health and its association with other dimensions of health and well-being and thus assist in the development of holistic eye care services and contribute to healthy ageing in India.

## Introduction

 The defining feature of the 21st century is population growth, accompanied by increased longevity and ageing. The global population is expected to peak at 10 billion in 2080, up from 8.2 billion in 2024.^1^ With a population of more than 1.4 billion in 2024, India is the most populous country in the world.^2^ The elderly or older people, defined as individuals aged 60 years and older, comprise about 10% of the population.^3^ This proportion is expected to increase to 20% or one-fifth of the total population.^1^ Ageing is a complex and multidimensional phenomenon often associated with a higher prevalence of systemic and ocular morbidities, including vision impairment (VI).^4 5^ VI affects more than a billion people worldwide.^6^ It is disproportionately higher in older populations, with more than 70% of those affected being 50 years and older.^6^ After age 50, VI increases significantly with each decade, reaching more than 75% among those aged 90 and older.^6^ The number of people with VI is projected to cross the 1.7 billion mark by 2050.^6^ Rising longevity, with its concomitant ageing population, mainly drives this increase from the estimated 1.1 billion today, with most of the increase occurring in low- and middle-income countries such as India.^6^ A recent study found that in a subregion of India (the state of Telangana), one out of every three older people had vision loss, 90% of which was attributed to avoidable causes.^7^ VI in the older people may lead to falls and accidents, resulting in fractures and adversely impacting their quality of life.^8^,^11^ Over 80% of VI can be managed with simple interventions like spectacles or cataract surgery.^12^,^16^

In addition to vision loss, other non-communicable diseases and disabilities are common in older age groups.^4 5^ These comorbidities and vision loss tend to cumulatively impact the daily functioning and overall quality of life in older individuals.^17^,^19^ Understanding how ocular and systemic health interact and influence overall well-being is essential for developing effective strategies that promote healthy ageing. In India, the impact of VI on quality of life and the effects of interventions on various factors such as cognition, depression, falls, fear of falls, multimorbidity and mortality in the older people have not been thoroughly studied. Also, the annual incidence of vision loss and the related risk factors among older people are not frequently reported. The Andhra Pradesh Eye Disease Study (APEDS), which encompassed the states of Telangana and Andhra Pradesh, reported on the incidence of VI across all age groups; however, the study included very few participants in the older age groups.^20^ Data from other countries cannot be extrapolated to the Indian setting due to diversity in socioeconomic development, cultural differences, public policies and the population profile.

Major comprehensive eye studies conducted in India include APEDS (1996–2000),^21^ Aravind Comprehensive Eye Study (1995–1997)^22^ and Central India Eye and Medical Study (2006–2008).^23^ These studies are cross-sectional in design and focused predominantly on eye health. Although these studies provided comprehensive information on vision loss and its associations, they had a limited focus on other comorbidities and disabilities and did not report on falls, fear of falling and the interactions between eye health and other disabilities, such as dual sensory loss or combined hearing and vision loss. Moreover, most studies either included participants of all ages (such as APEDS) or those aged 40 years or older, thereby limiting the number of people in older age groups in the sample. No major population-based studies reported on the impact of eye interventions such as spectacles and cataract surgery, or on other dimensions of health and well-being among community-dwelling older people in India.

The previous longitudinal Hyderabad Ocular Morbidity in Elderly Study (HOMES) provided vital insights into the burden and causes of vision loss among the older people in the Hyderabad region in Telangana.^24^,^26^ However, as HOMES included only the people in residential care in an urban area, the results cannot be generalised to the general population. Additionally, the small sample size prevented adequate assessment of the factors related to VI incidence and their association with other systemic and cognitive risk factors. Because the number of older people in India is increasing dramatically and is expected to reach 323 million by 2050, data on vision loss and its correlates are essential for planning eye care programmes.^27^ Addressing vision loss is vital as it has complex and far-reaching consequences on the overall health and well-being of the older people.

These gaps in the literature highlight the need for a more comprehensive longitudinal study of eye health among the older people. This study should include the examination of other disabilities, multimorbidity, the effects of interventions and the incidence of vision loss in India. Additionally, evaluating how vision loss affects daily activities among the older people in the community is important. Conducting such a holistic ‘eye health and ageing’ study can serve as a foundation for creating older people centric comprehensive eye health programmes in India.

The Longitudinal Eye Health, Ageing and Disability Study (LEADS) is an eye health study that extends to other dimensions of health and well-being in the community-dwelling older adults of the two predominantly Telugu-speaking states of Andhra Pradesh and Telangana in southern India, which have a combined population of about 92 million in 2025. LEADS has the following objectives: (a) to determine the prevalence, causes, risk factors and impact of VI in the older people in both states; (b) to understand the associations between VI and sociodemographic risk factors, systemic health factors (multimorbidity), physical performance, hearing, cognitive function, social connectedness and other factors among the older people; (c) to assess the impact of eye interventions such as cataract surgery and refractive correction (for distance and near) on cognitive function, depression, physical performance, falls, fear of falling, quality of life and visual functions; and (d) to assess the annual incidence of and risk factors for VI among the older people in the community. This paper describes the objectives, methods and examination protocols LEADS follows.

## Methods and analysis plan

### Patient and public involvement

Patients and/or the public are not involved in the design, conduct, reporting or dissemination of the findings from this research.

### Study setting

**Figure 1:**
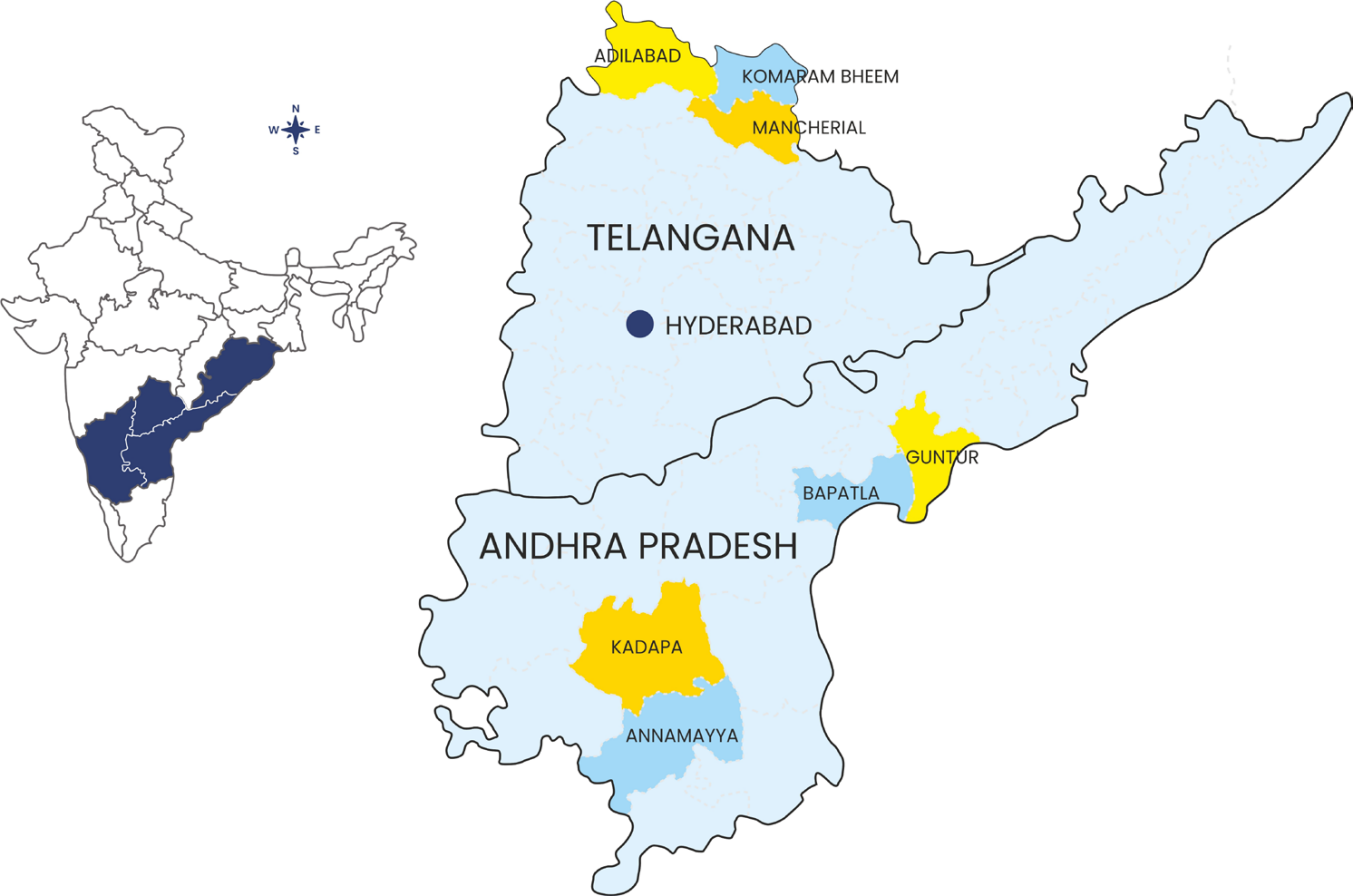
Map showing the study area.

LEADS is a longitudinal, population-based epidemiological study of individuals aged 60 years and older living in two predominantly Telugu-speaking states, Andhra Pradesh (including coastal Andhra Pradesh and Rayalaseema) and Telangana (the former combined state of Andhra Pradesh), with a total population of approximately 84 million, according to the 2011 census and a projected population of 92 million in 2025. In total, seven districts across three regions are included: Guntur and Bapatla in coastal Andhra Pradesh; Kadapa and Annamayya in Rayalaseema; and Adilabad, Mancherial and Kumuram Bheem Asifabad in Telangana (figure 1). The combined population of these seven districts is about 10 million, based on the 2011 census. Guntur and Bapatla in coastal Andhra Pradesh are economically well-developed areas with fertile land, flourishing agriculture and higher literacy levels. On the other hand, Kadapa and Annamayya in Rayalaseema represent a dry, arid, drought-prone rain-shadow region. Adilabad, Mancherial and Kumuram Bheem Asifabad in Telangana represent predominantly rural and tribal regions with lower population density and lower literacy. Together, these 7 districts represent the two states of Andhra Pradesh and Telangana (the erstwhile combined state of Andhra Pradesh).

Two examination waves (wave I and wave II) are planned to occur 18–24 months apart, with wave III planned for the future. The follow-up interval is planned at 18–24 months, based on prior prospective studies assessing the impact of major interventions, such as cataract surgery and spectacles for uncorrected refractive errors and near-VI. The time interval between preintervention and postintervention in these prospective studies ranged from 2 months to 18 months for cataract surgery^28^,^36^ and for URE and presbyopia from a few weeks to 1 year.^37^,^41^

### Sample size and sampling process

The sample size for LEADS was based on the two large studies conducted in the region. APEDS reported a 5% annual incidence of VI (defined as presenting visual acuity (PVA) worse than 6/12 in the better eye) in the older age group.^20^ The incidence of VI at the end of 8 months in HOMES was 6%. Based on these two studies, using a conservative estimated annual incidence of 3% VI, a precision of 20% of prevalence, a 95% CI, a design effect of 1.6 to account for a cluster size of 60 participants and a 15% non-response rate, the sample size needed is 5827 (rounded to 6000). Assuming a 20% loss to follow-up at wave II, the final sample size required is 7200 (120 clusters). This sample size is also adequate to estimate a 10% change in visual function scores, EuroQol-5D and other scores after the intervention. With this sample size, the study is adequately powered for subgroup analyses of VI risk factors and to analyse the association with other dimensions of health and well-being.

A two-stage cluster random sampling method is used. In the first stage, clusters are systematically sampled using probability proportional to size from a sampling frame that includes all census villages. In each cluster, 60 individuals aged 60 and older are selected through the compact segment sampling method and door-to-door enumeration. These 60 individuals are invited to participate in the study. If individuals are unavailable on the first visit, at least two attempts are made to contact them. All individuals examined in wave I (baseline) will be followed up with after 18–24 months during wave II.

### Inclusion/exclusion criteria

All participants aged 60 years or older and living in the selected household for at least 1 month prior to the day of enumeration are included. The age of the participant is verified with one of the government-issued identity cards, and/or by connecting the personal milestones to historical events such as Independence Day.^42^ The participants who are homebound due to physical/mental disabilities or old age are included in the enumeration, but a shorter version of the study protocol is used as a home consultation. This includes personal and demographic information and systemic health and eye examinations, which are considered a common minimum dataset. Individuals aged less than 60 years, guests/visitors living in the household for less than a month at the time of enumeration are not included.

### Study instruments

LEADS has two study components: a nonclinical component (questionnaire-based interviews) and a clinical assessment (a clinical eye examination). The nonclinical component is always completed before the clinical component. A few of the clinical and nonclinical assessment protocols were used in a previous study with the elderly population.^25^

#### Individual characteristics, lifestyle, systemic health and disabilities

- *Personal, demographic and family information*: age, gender, years of education, marital status (unmarried/married/single/widowed), previous/present occupation, family structure, current income, sources of revenue and languages known (spoken and written) are documented. Asset scoring is done using an equity tool that comprises a set of questions to assess wealth equity.^43^ Daily time-use patterns are also evaluated using a questionnaire.
- *Self-report of systemic history and medication*: the history of systemic health conditions such as hypertension, diabetes, heart disease and other conditions is recorded, including their duration and current medications. Access to healthcare and healthcare utilisation are assessed using a questionnaire.
- *Lifestyle factors*: information on smoking and alcohol consumption (present and past use) and exposure to ambient air pollution due to household fossil fuels is assessed. Both the current status and past exposure to ambient air pollution fuels are documented.
- *Blood pressure*: blood pressure is measured using the Omron digital blood pressure apparatus (OMRON Healthcare India).
- *Body mass index*: height and weight are recorded to compute body mass index.
- *Cognitive function*: Hindi Mini-Mental State Examination (HMSE) is used for assessing cognitive function.^44^ HMSE is a modified version of the Mini-Mental State Examination.^45^ Items that require reading, writing and arithmetic skills were modified in HMSE for rural populations. It consists of 22 items or questions that assess 10 key cognitive areas, including orientation to time and place, registration, attention, concentration, object recognition, language abilities, both understanding and expressive speech, motor skills and praxis.^44^
- *Depression*: depressive symptoms are assessed using the Patient Health Questionnaire (PHQ-9).^46^,^48^
- *Hearing*: hearing ability is assessed using the Hearing Handicap Inventory for the Elderly Screening questionnaire^49^ and whisper test.^50 51^ The whisper test includes recalling three of six words whispered by an examiner from behind the participant. Each ear is tested separately. This test has been shown to have good sensitivity and specificity for screening for hearing loss.^50 51^
- *Physical functioning*: physical functioning is assessed by performing the Short Physical Performance Battery (SPPB).^52^ SPPB includes gait speed, chair stand test and balance.^52^
- *Visual functioning*: visual functioning is assessed using a 17-item questionnaire validated for the Indian population and used in earlier studies.^53 54^
- *Fear of falling and falls*: the Falls Efficacy Scale questionnaire is used to assess fear of falls. The history of falls in the last year, along with the consequences of the falls, is recorded as described in previous studies.^55 56^
- *Disability and assistive devices*: the WHO Disability Assessment questionnaire (WHO-DAS) is used to assess functioning in six domains covering mobility, cognition, self-care, leisure activities and social interactions. Information on the use of assistive devices is also collected.
- *Nutrition and oral health*: Mini Nutritional Assessment-Short Form is used to assess nutritional status.^57 58^ To assess oral health status, three questions are asked. These include the ability to chew food such as chapati and guava, loss of natural tooth and an open-ended question to document other oral health issues.
- *Sleep pattern*: two questions are asked to determine sleep difficulties and possible reasons for them. This covers trouble falling asleep, staying asleep or waking up unrefreshed in the past 30 days, along with potential reasons for any reported issues.

#### Social connectedness, quality of life and well-being

- *Health-related quality of life*: EuroQOL 5D-5L (EQ5D) is used to assess the health-related quality of life. EQ5D encompasses five dimensions: mobility, self-care, usual activities, pain/discomfort and anxiety/depression.^59 60^ The Telugu version of the questionnaire is available and used after obtaining permission from the developers. EQ5D values will be used to assess the quality-adjusted life years for cost-utility analyses.^59 60^
- *Health and well-being*: subjective psychological well-being is assessed using the WHO Well-Being Index questionnaire.^61^
- *Satisfaction with life*: life satisfaction is assessed using the Satisfaction with Life Questionnaire.^62^
- *Instrumental activities of daily living/activities of daily living (IADLs/ADLs)*: IADLs/ADLs are assessed using Lawton’s IADL scale and Katz’s Index of Independence in ADLs, respectively.^63 64^
- *Social networking and connectedness*: the participant’s social connections and societal roles are assessed using the Social Networking Index (SNI) questionnaire.^65^
- *Physical activity*: physical activity, including time spent on vigorous and moderate activity and walking activities, is assessed and quantified using the International Physical Activity Questionnaire.^66^
- *Verbal autopsy questionnaire*: this questionnaire is administered at the annual in-person follow-up visits to the family members to collect mortality-related data of deceased study participants during the follow-up period.^67^

#### Clinical eye examination

The clinical assessments start with an evaluation of measures of frailty (hand grip strength using the Digital Grip Dynamometer, Camry model EH101, Camary Industries, Hong Kong) and the SPPB.^52^ A gait speed test (3-metre distance) is followed by measurements of height, weight and blood pressure, and performance-based measures, all conducted by a trained vision technician.^68^

- *Distance visual acuity* (DVA): DVA is measured with the standard logMAR (logarithm of the minimum angle of resolution) tumbling ‘E’ chart or English alphabet charts. Visual acuity (VA) is tested with the subject’s current refractive correction if used. Unaided VA is recorded in all cases, and pinhole VA is recorded if unaided VA is worse than 6/9 (log MAR 0.2). The standard Snellen chart is for home examinations.
- *Near vision*: Near VA (NVA) is measured at a distance of 40 cm using the logMAR near vision ‘E’ chart with the current refractive correction, if any. Unaided NVA is assessed in all cases. Both monocular and binocular near vision are assessed. An N-notation chart is used for home examinations.
- *Contrast sensitivity*: contrast sensitivity is measured with the Berkeley Discs Contrast Sensitivity Test (Precision Vision, Inc., Woodstock, IL, USA) using habitual spectacle correction at 50 cm. This test features double-sided cards with a six-cell grid with three cells containing 50 mm circles and three empty cells. This test is easy to administer to older people with low literacy.
- *Stereopsis (depth perception)*: stereopsis is measured using a random-dot stereogram (Stereo Optical Co., Inc., Chicago, IL, USA).
- *Colour vision*: colour vision is assessed using Hardy-Rand-Rittler test plates at a distance of 50 cm with glasses.^69^
- *Performance-based measures (functional vision)*: performance-based measures (functional vision assessment) are evaluated using computer-based tests that include face recognition (modified common faces recognition test),^70^ facial expression recognition and visual search tasks.^71^ The accuracy and time taken to complete the task are documented.^25 72^
- *Slit lamp examination/anterior segment imaging*: slit lamp examination is conducted using a handheld portable slit lamp biomicroscope (Keeler PSL Classic Portable Handheld Slit Lamp, UK). The eyelids, conjunctiva, cornea, anterior chamber and lens are examined and documented as either normal or abnormal, with the abnormality specified. Anterior segment imaging is performed using the Remido Portable Digital Slit Lamp (Remido PSL D20, Remidio Innovative Solutions Pvt. Ltd., Bangalore, India) to document anterior segment abnormalities and ocular morbidity.
- *Refraction*: refraction (manual and auto-refraction) is performed for all participants with presenting DVA worse than 6/9 or near vision worse than N6 in either eye. The best-corrected distance and NVA are obtained and documented. Auto refraction is performed using the E-See portable auto refractor (PlenOptika, Inc., USA; Aurolab, Madurai, India).
- *Intraocular pressure (IOP) measurement*: IOP is measured using a non-contact tonometer (iCare IC100; Icare Finland Oy, Vantaa, Finland).
- *Fundus examination and imaging*: fundus examination and imaging are done using a non-mydriatic fundus camera (Remedio Fundus on Phone (FOP NM-10), Remidio Innovative Solutions Pvt. Ltd., Bangalore, India). This camera features an offline artificial intelligence algorithm that detects glaucoma, age-related macular degeneration and diabetic retinopathy.^73 74^
- *Assessment of dry eye*: the Ocular Surface Disease Index questionnaire is used to assess the frequency and severity of the symptoms of dry eye.^75^ Additionally, tear breakup time (TBUT) and Schirmer’s tests are performed.^76^ Schirmer’s test uses a 35×5 mm filter strip placed laterally in the lower fornix to measure the volume of tears within 5 min without anaesthesia. If the wetting of the strip is less than or equal to 10 mm, then it is considered suggestive of dry eye. TBUT is assessed using sterile fluorescein strips. The participants are instructed to look upward, then the strip is placed at the conjunctival inferior fornix, and finally, participants are advised to blink. The tear film is observed under a cobalt blue filter for the appearance of dry spots. The time interval between the last blink and the first appearance of the dark of the dry spot is recorded. A TBUT of less than 10 seconds is considered suggestive of dry eye.^76^

### Data collection process

**Figure 2:**
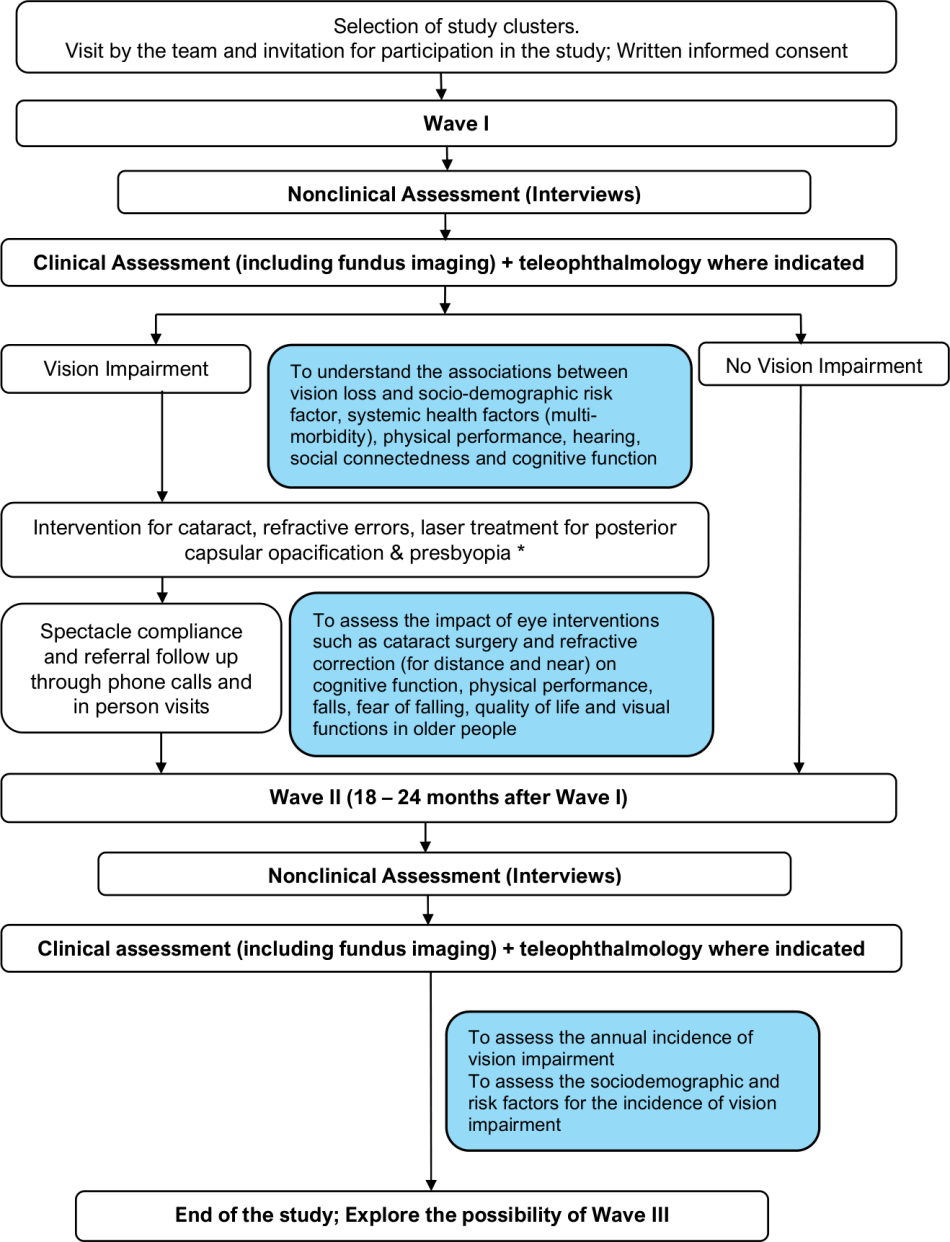
Flow chart showing the Longitudinal Eye Health, Ageing and Disability Study processes. *Participants with vision loss or any other eye health issues will be provided with services free of cost at L V Prasad Eye Institute centres or through using teleopththalmology.

Two teams, each consisting of at least one optometrist, one vision technician and three field investigators, collect data. These teams work in parallel across different clusters, supervised by a project coordinator who provides logistical support and is responsible for micro-planning data collection and getting local approvals for clinic setup. The following four-step study process is followed for LEADS at the baseline (wave I). The same data collection process is followed for the follow-up visit (wave II), except for step 1 (figure 2).

#### Cluster selection and enumeration

The study team visits the selected households in the randomly selected segment in the villages (clusters). The field investigators conduct door-to-door enumeration (listing) to identify eligible participants and obtain consent.

#### Interviews at the residence

After obtaining consent, trained field investigators conduct the interviews in person at the households of the selected participants in the local language (Telugu/Hindi). Interviews are audio-recorded with the participants’ consent and reviewed as part of the quality control process. For questionnaires not available in regional languages, standard translation guidelines, including forward translation, reconciliation of differences and backward translation by bilingual translators, are followed.^77^ Adequate breaks are provided to participants during the interview process.

#### Clinical assessment at the makeshift clinics

After completion of the interview, an appointment is scheduled for the clinical examination at the makeshift clinic in the vicinity. Typically, this clinic is set up in a primary health centre, a gram panchayat office or other government facilities, such as schools. A checklist is used to select the clinic sites, including elderly-friendly pathways, a reliable power supply and adequate ambient lighting for clinical assessments. A light metre is used to ensure a minimum of 200 lux in the clinic set-up. The clinical competence of vision technicians in conducting eye examinations is published in the literature.^78 79^

#### Intervention and compliance assessment

After the clinical assessment, spectacles are provided for all participants who need them. Participants are asked to select the spectacle frame of their choice from the inventory of frames available at the makeshift clinic. The spectacles are made to order and delivered to the households within 2 weeks. Spectacle compliance is assessed every 6 months after provision through a telephone interview or a physical visit to households, using a structured questionnaire. Participants with VI (excluding refractive errors) or those requiring eye care services are referred to the nearest L V Prasad Eye Institute eye care centre for management. A referral letter is provided for the participants, and appointments for the visit are arranged. All services are provided at ‘no cost’ to the participants. All individuals referred for cataract surgeries are followed up within 6 months to ensure that surgical services are used. Assistance, such as free transportation, is provided if required to encourage the uptake of services. For those who fail to use the services, a questionnaire is administered to understand the reasons for not utilising them. Teleophthalmology services are provided wherever possible.

In addition, annual in-person visits were conducted in each participant’s household, during which data on uptake of eye care services and compliance with spectacle use were assessed. In cases of mortality, the information on the possible cause of death was obtained from the family members and documented. These initiatives are intended to help maintain contact with the participants between the two waves of the study. Participants who require additional health interventions are referred to the nearest primary care centre or a local medical practitioner for management. In addition to maintaining regular contact with the participants, the provision of complete eye examinations at the doorstep and free services is envisaged to remain a motivating factor for participation in the wave II study. All participants examined in wave I are eligible for the examination in wave II. Both the nonclinical and clinical assessments will be conducted as in wave I. The verbal autopsy questionnaire will be used to ascertain the causes of death for the participants who died between waves.^67^

### Case definitions

- *Distance VI (DVI)*: DVI is defined as presenting visual acuity (PVA) worse than 6/12 in the better eye, as defined by the WHO.^80^ This is further subdivided into mild VI (PVA worse than 6/12 to 6/18), moderate VI (PVA worse than 6/18 to 6/60), severe VI (PVA worse than 6/60 to 3/60) or blindness (PVA worse than 3/60 to no light perception).^80^
- *Avoidable VI*: VI due to cataracts, uncorrected refractive errors or posterior capsular opacification is considered avoidable VI.
- *Incidence of VI*: the incidence of any VI is defined as ‘new cases’ of VI among those who had no VI at the baseline (wave I).
- *Progression of VI*: progression of VI is defined as worsening of VI by at least one WHO category in the better eye from the baseline (wave I) to the follow-up visit (wave II).
- *Non-visually impairing ocular morbidity*: any eye condition or ocular abnormality without VI but resulting in ocular symptoms and discomfort is considered non-visually impairing ocular morbidity. Examples of ocular morbidity include ocular surface disorders such as dry eye, meibomitis, blepharitis, lid anomalies and pterygium.
- *Near VI (NVI)*: presenting near vision worse than N6 in the better eye, but no DVI is considered of NVI.

The principal cause of VI is documented for each eye and then for the person. If multiple causes exist, the most easily treatable or correctable one is identified as the primary cause of VI for that individual.

### Data management

Data collection is conducted in the field using digital forms within a specifically developed application on digital tablets and paper forms. Data are synced to an encrypted secure server after every interview and clinical assessment. When a network signal is unavailable, the application operates offline and synchronises data with the cloud as soon as a signal is detected. The data manager accesses and downloads the data daily to generate a summary report, ensuring that all data are synced. A unique identification number is generated for each participant and is common across all databases. Data analysis is conducted using Stata statistical software for Windows, V.17 (StataCorp, College Station, Texas, USA).^81^ Only deidentified datasets will be used for analysis. The prevalence estimates will be adjusted to the age and gender distribution of the population and will be presented with 95% CIs.

A reliability study was conducted prior to the main study, and an assessment was performed to evaluate interobserver agreement on VA and clinical examination results. This assessment compared the optometrists and vision technicians who conducted the tests with a ‘gold standard’ senior optometrist. The inter-examiner reliability among the field investigators for all the questionnaires is assessed. A kappa statistic (for categorical items) or an intraclass correlation coefficient (for continuous measures) is calculated as appropriate using Stata statistical software, V.17. An intraclass correlation coefficient or kappa statistic of at least 0.7 is considered acceptable for all scores obtained with questionnaires.

### Statistical analysis plan

The primary analysis at the baseline (wave I) will focus on VI and its risk factors and causes. The demographic associations of VI with prevalence will be assessed using multiple logistic regression models and adjusted ORs with 95% CIs will be reported. The regression models will be evaluated using variance inflation factors and goodness-of-fit tests. An analysis of variance and t-tests will be conducted to assess the mean differences in visual analogue scale (VAS) and EuroQol-5D scores among VI subgroups. The visual function scores and EuroQol-5D scores of individuals with and without VI will be compared. Similarly, other scores of individuals with and without VI, such as the Satisfaction with Life Scale, HMSE, PHQ-9, SNI, WHO Well-Being Index and the WHO-DAS, will be compared. The secondary analysis will include compliance with spectacle use and the uptake of referral services.

The primary analysis at the follow-up (wave II) will focus on incidence (new cases of VI), progression (worsening of VI) and associated factors. Comparisons will be made for each participant between the baseline (wave I) and the follow-up assessment (wave II). In addition, among those who received interventions such as spectacles, laser capsulotomy and cataract surgery at baseline (wave I), the impact of these interventions on visual functioning and other secondary outcomes, including cognition, social connectedness, falls, fear of falling, activities of daily living and other measures, will be assessed. Preintervention and postintervention mean scores will be compared using a Student’s t-test (for continuous variables) in univariable analysis. Wilcoxon signed-rank test (for ordinal data) and McNemar’s test (binary data) will be used. The analysis of covariance test will be used to compare the means of continuous dependent variables, controlling for other covariates. In this, the postintervention scores will be used as an outcome variable and baseline scores as a covariate, adjusting for baseline differences between the groups. In addition, difference-in-differences logistic regression will be used to assess the impact of interventions (cataract surgery and spectacles).^82^

### Pilot study

A pilot study was conducted with 203 participants to field test the study protocols and fine-tune the workflow. The interview duration ranged from around 30 to 60 min excluding the break in between depending on the responses from the participants. The clinical examination lasted for around 40–60 min. Learning from the pilot study improved field-level planning, logistics and assessment workflow for the main study. Data collection for the main study started in June 2024 and is currently underway. It is scheduled to be completed by May 2027.

### Expected outcomes

LEADS is designed to provide valuable data and a solid evidence base for planning eye care services for the older people in two large states in southern India.

The major strengths of LEADS are the large sample sizes from three locations representing an estimated population of about 92 million people in the two states of Andhra Pradesh and Telangana in Southern India. Establishing clinics closer to communities is expected to improve response rate and increase convenience for the older people by easing the burden of travel. The inclusion of home visits for homebound individuals is an added strength of this study. The use of standardised study protocols by well-trained teams adds robustness to the protocol and data collection. As ‘no cost’ service delivery is an integral part of the study, greater support and response from the local authorities and communities are expected. In addition, having regular contact with participants is expected to help maintain a good response rate in the follow-up waves. Despite all efforts, it is likely that there could be differential participation. It is plausible that individuals who are healthier, more educated or of higher socioeconomic status may be more likely to participate compared with others. The efforts are being made to maintain a high response rate to minimise this inherent healthy cohort bias in this longitudinal study.

LEADS is intended to supplement data from ageing studies, such as the Longitudinal Ageing Study in India (LASI), and provide insights into the relationships among multimorbidity, VI and overall well-being of the older people in the community.^83^ In addition, the study will provide valuable data and form a basis for developing holistic eye care services to ensure that the older people do not suffer from needless vision loss in their sunset years. Thus, eye care as an entry point to holistic healthcare could become a possibility. After all, healthy ageing is happy ageing.
